# Population reach and participation rate in opportunistic vs. systematic atrial fibrillation screening: STROKESTOP III

**DOI:** 10.1093/europace/euag214

**Published:** 2026-08-30

**Authors:** Mashroor Khan, Sofia Skröder, Michael Ingre, Fredrik Carlstedt, Anders Eriksson, Johanna Star Tenn, Mårten Rosenqvist, Emma Svennberg

**Affiliations:** Department of Medicine Huddinge, Karolinska Institutet, Karolinska University Hospital Huddinge, Stockholm SE 141 86, Sweden; Centre for Clinical Research and Education, Region Värmland, Karlstad, Sweden; Faculty of Medicine and Health, School of Medical Sciences, Örebro University, Örebro, Sweden; Department of Medicine Huddinge, Karolinska Institutet, Karolinska University Hospital Huddinge, Stockholm SE 141 86, Sweden; Centre for Clinical Research and Education, Region Värmland, Karlstad, Sweden; Department of Clinical Physiology, Region Värmland, Karlstad, Sweden; Department of Clinical Physiology, Region Värmland, Karlstad, Sweden; Department of Clinical Sciences, Danderyd University Hospital, Karolinska Institutet, Danderyd, Sweden; Department of Medicine Huddinge, Karolinska Institutet, Karolinska University Hospital Huddinge, Stockholm SE 141 86, Sweden

**Keywords:** Atrial fibrillation, Screening, Stroke, Oral anticoagulants, Opportunistic screening

## Abstract

**Aims:**

Population-based screening for atrial fibrillation (AF) should be considered in elderly individuals to enable AF detection. However, participation in systematic screening programmes is often suboptimal. STROKESTOP III evaluated whether opportunistic screening could improve participation compared with systematic screening.

**Methods and results:**

STROKESTOP III is a cluster-randomized trial including individuals aged 75–76 years from 16 primary care centres in Region Värmland, Sweden. Centres were randomized to systematic screening, using mailed invitations, or opportunistic screening, using invitations during visits.

In the systematic arm, 1312 individuals were eligible and invited, of whom 607 participated (46.3%). In the opportunistic arm, 1390 individuals attended participating primary care centres and were potentially eligible for invitation. However, 641 individuals (46.1%) were not assessed for eligibility. Among the 749 assessed individuals, 609 were eligible and 374 participated.

Participation among invited eligible individuals was significantly higher with opportunistic than systematic screening (374/609, 61.4% vs. 607/1,312, 46.3%; *P* < 0.005). However, overall reach was lower in the opportunistic arm because of incomplete assessment for invitation (374/1,479, 25.3% vs. 607/1,437, 42.2%; *P* < 0.005). Participants in the opportunistic arm had a higher cardiovascular risk burden, including more hypertension, diabetes, and a higher CHA_2_DS_2_-VASc-score (3.99 vs. 3.60; *P* < 0.005). Exclusion rates were higher (18.7% vs. 8.7%; *P* < 0.005), mainly due to previously diagnosed AF and cognitive impairment.

**Conclusion:**

Opportunistic screening increased participation among invited individuals and identified a higher-risk population, but its overall population reach was limited by incomplete eligibility assessment. Combining opportunistic and systematic strategies may optimize reach and participation in AF screening programmes.

## Graphical Abstract

**Graphical Abstract:**
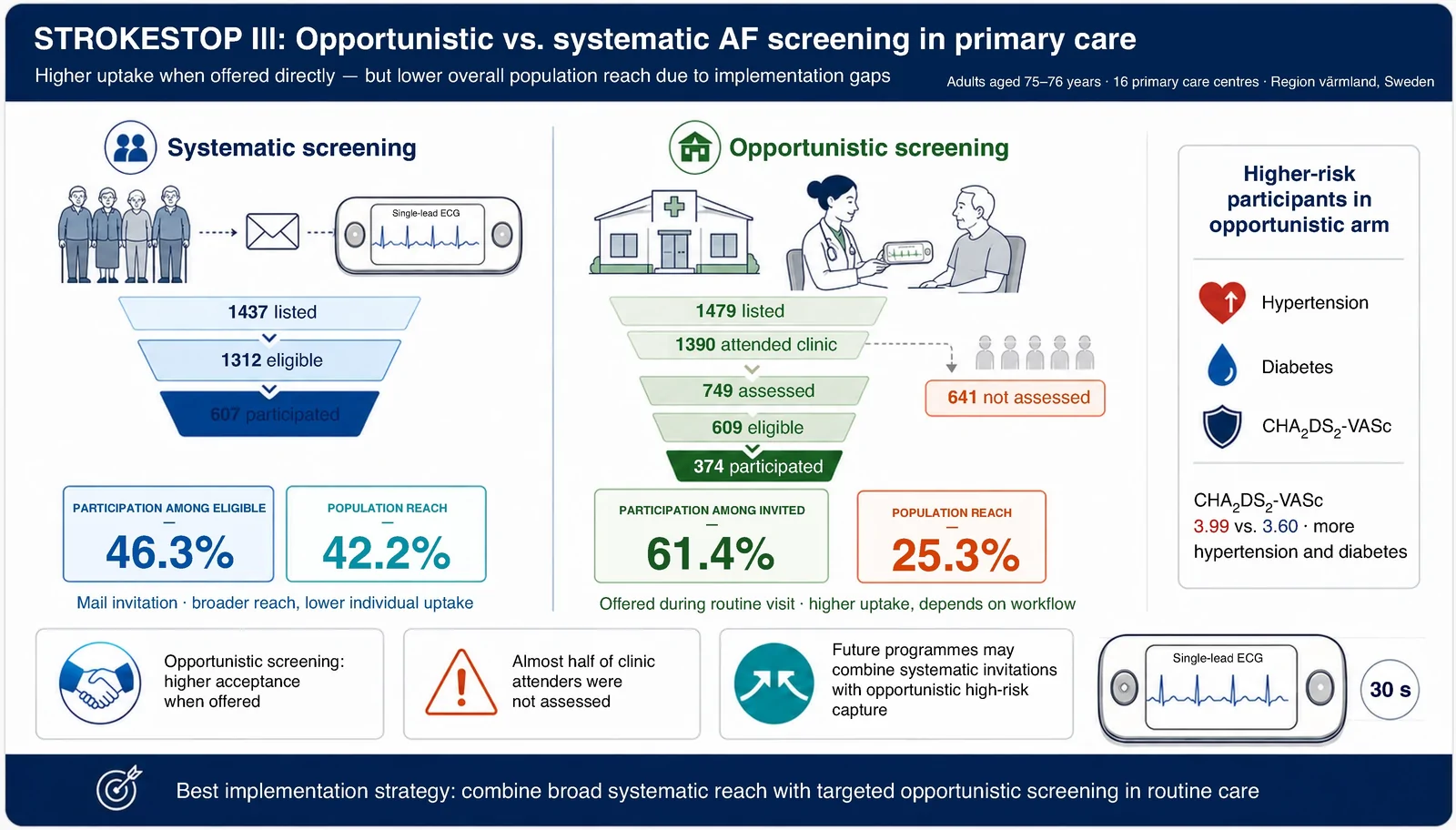

What’s new?

- STROKESTOP III is the first cluster-randomized study directly comparing opportunistic and systematic atrial fibrillation screening strategies in routine primary care.
- Opportunistic screening achieved significantly higher participation among eligible invited individuals compared with systematic population-based screening (61.4% vs. 46.3%).
- Despite higher uptake among those actively offered screening, opportunistic screening achieved lower overall population reach (25.3% vs. 42.2%) because many individuals attending primary care were not assessed for eligibility.
- Participants recruited through opportunistic screening had a higher cardiovascular risk burden and higher CHA_2_DS_2_-VASc-scores.
- The study highlights important real-world implementation challenges associated with opportunistic atrial fibrillation screening in primary care.
- Combining systematic invitations with opportunistic screening may optimize both participation and population reach in future atrial fibrillation screening programmes.

## Introduction

Atrial fibrillation (AF) affects a large portion of the elderly population and increases the risk of heart failure, stroke, and dementia.^1–3^ Around one-third of AF cases may be asymptomatic and hence undiagnosed and untreated.^4^ Recent global analyses from the Global Burden of Disease project have demonstrated a substantial increase in the worldwide burden of AF over the past decades, with projections suggesting a continued increase in prevalence and burden on healthcare in the coming decades.^5,6^ Early diagnosis of AF and prompt initiation of treatment with oral anticoagulants (OAC) can reduce stroke risk by up to 70%.^7^ In the 2024 European Society of Cardiology’s guidelines, screening for AF through routine heart rhythm assessment during a healthcare visit got a Class I recommendation for individuals aged above 65.^8,9^ The STROKESTOP I trial showed that systematic AF screening can increase AF detection, reduce the risk of a combined endpoint including stroke, death and bleeding, and be economically effective.^10,11^ Overall, AF screening studies employing a population screening strategy have been affected by a low participation rate of 50%.^10,12^ Those choosing to participate in the trials generally had fewer risk factors for stroke and AF compared with non-participants. Previous AF screening studies have suggested that opportunistic screening, where individuals are invited during routine healthcare encounters, may improve participation rates.^13,14^ In many settings, opportunistic screening refers to pulse palpation or single-time-point rhythm assessment during a healthcare encounter commonly limiting detection of paroxysmal AF.^15^ However, in the present study, opportunistic screening refers to the recruitment method rather than the duration of the ECG screening. For both groups, the screening consisted of a repeated handheld ECG recording over 2 weeks. Findings from the two decades old SAFE study further indicate that opportunistic screening is comparable to systematic screening in terms of AF detection, while offering greater cost-effectiveness.^16^

The STROKESTOP III study aimed to investigate whether opportunistic screening could be used to increase the participation rate compared with population screening.

## Methods

### Study population and invitation procedure

The study design has previously been published.^17^ To summarize, individuals born in 1948/1949 (*n* = 2916) residing in the catchment area of 16 primary care centres in Region Värmland, Sweden, were invited to the study. The centres were stratified using cluster randomization, considering size and sociodemographic factors.

**Figure 1:**
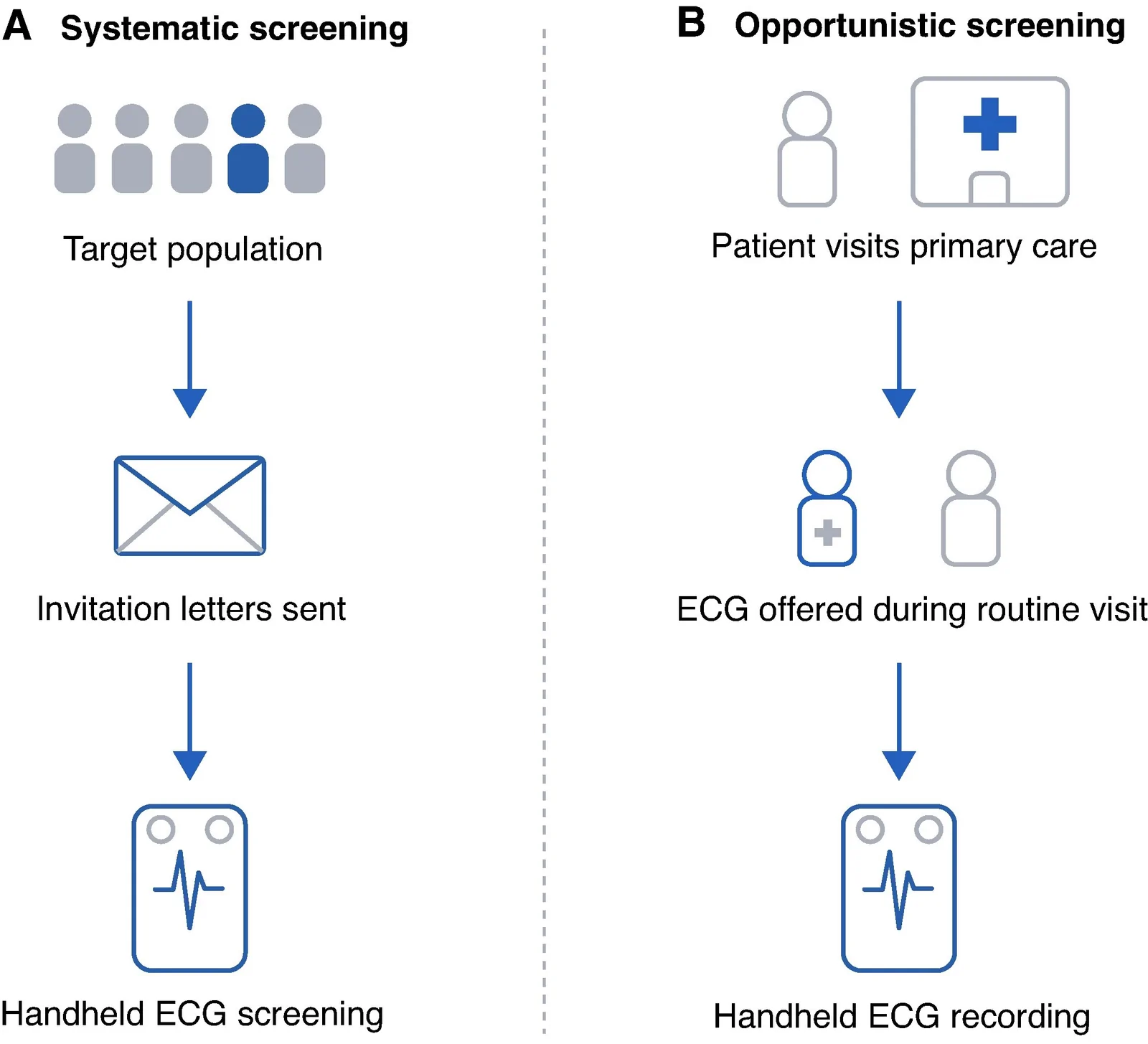
Flowchart of comparison between opportunistic screening compared to systematic screening.

For primary care centres allocated to the systematic arm, participants received an invitation via mail, with one additional reminder sent to non-responders. In the opportunistic arm, participants were identified and invited during a regular healthcare visit, or subsequently by phone if there was insufficient time during the visit (*Figure 1*). Opportunistic screening was performed by the regular clinic staff after training, but without additional personnel or external support.

Exclusion criteria included known AF, ongoing OAC treatment, and cognitive impairment. These criteria were verified by the inviting centre in the opportunistic arm. In the systematic arm, verification was primarily based on self-reported health declarations, with medical records reviewed when necessary to resolve uncertainties.

In the opportunistic arm, non-assessment was defined as an individual allocated to the opportunistic arm, born in 1948/49, who attended the primary care centre during the study, but who was not assessed for participation regardless of eligibility.

Eligibility assessment included identifying the individual as part of the study population, checking for exclusion criteria, confirming their practical ability to perform home ECG recordings, providing study information, and inviting the individual to participate if these criteria were fulfilled.

### Screening procedure

Individuals in the opportunistic arm were invited verbally followed by written information. In the systematic arm, written information was mailed to those eligible. All participants were asked to sign informed consent forms prior to study participation. In addition, participants were asked to provide information regarding comorbidities, including thromboembolic risk factors to calculate the CHA_2_DS_2_-VASc-score, chronic obstructive pulmonary disease (COPD), valvular disease, hyperthyroidism, chronic kidney disease (CKD), rheumatoid arthritis (RA), diabetes, hypertension, vascular disease, and gout. Participants were also asked about any ongoing treatment with OAC and a history of symptomatic palpitations.

Participants were instructed to perform a 30-second single-lead handheld ECG three times daily for 2 weeks and when experiencing any palpitations using Zenicor One (Zenicor Medical Systems, Stockholm, Sweden). The Zenicor One device has been used in several previous studies and has demonstrated high accuracy for AF detection.^18^

All ECGs were reviewed by trained healthcare personnel with expertise in ECG analysis. When AF could not be confidently excluded, recordings were further assessed by a specialise physician. If AF was detected, the participant was referred to their primary care physician for treatment. When AF could not be excluded on the single-lead ECG or when recording quality during the 2-week study period was insufficient, an additional 2-day or 7-day Holter recording was performed to confirm the diagnosis.

### Statistical analysis

The sample size was based on the number of potentially eligible individuals at participating primary care centres (*n* = 93–405) and the expected availability of these individuals during the study period. Regional data indicate that >90% of 75-year-olds in Värmland attend primary care at least once annually; accordingly, 90% of eligible individuals were assumed to be available for recruitment. The primary endpoint in the study design paper was the participation rate. In the present report, we distinguish between participation rate and overall population reach to separate individual acceptance from implementation coverage. Participation rate was defined as the proportion of eligible and invited individuals who participated. In the systematic arm, this was calculated as participants among eligible individuals after exclusion criteria were applied. In the opportunistic arm, this was calculated as participants among individuals who were assessed for eligibility and actively offered screening. Overall population reach was defined as participants among all listed individuals in each randomized arm.

Expected participation rates were derived from STROKESTOP I and II, suggesting ∼50–55% participation with systematic screening. For opportunistic screening, a participation rate of 70% was estimated. Power calculations were performed to detect the difference between groups in a cluster-randomized design, assuming an intraclass correlation coefficient (ICC) of 0.03 and a two-sided alpha of 0.05.

Healthcare centres were added iteratively in pairs (one per study arm) until sufficient power was achieved. Inclusion of 16 centres (*n* = 2656 individuals) provided 82% power. Calculations were performed using the *clusterPower* package in R.^19^

The participants’ baseline characteristics, including sex and medical history including the risk factors, were summarized using frequencies for categorical variables and means with standard deviation (SD) for continuous variables. To evaluate differences between the groups, the χ^2^ test was used for categorical variables, and the Mann–Whitney *U* test was used for ordinal values. A *P*-value of <0.05 was considered significant. R statistical software (R foundation for Statistical Computing, version 4.5.3, Vienna, Austria) was used for statistical analysis.

### Ethics

The study complies with the Declaration of Helsinki, and the protocol was approved by the regional ethics committee in Stockholm (DNR 2023–04 659–01). Written informed consent was obtained from all participants in the screening programme.

## Results

**Figure 2:**
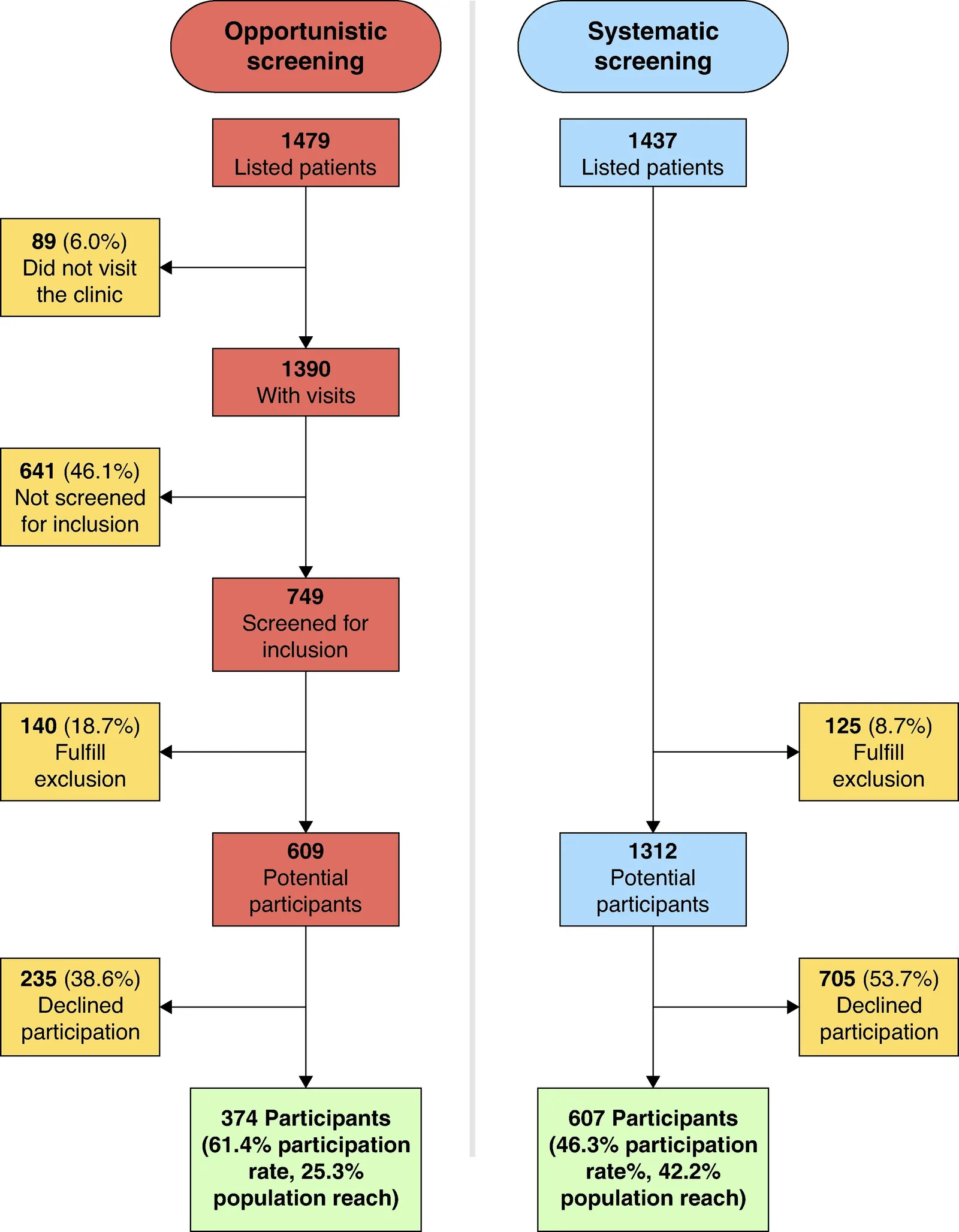
Flow diagram showing eligibility, fulfilment of exclusion criteria and participation rates in the opportunistic and systematic screening arms of the STROKESTOP III study. Participation rate refers to the number of individuals that accepted study invitation among invited individuals. The population reach refers to the number of individuals that accepted study invitation from the whole population.

The study was initiated in May 2024 and concluded in October 2025. In the systematic arm, 1437 patients were allocated to the eight primary care centres, as shown in *Figure 2*. A total of 125 (8.7%) of the participants fulfilled the exclusion criteria; hence, 1312 eligible participants remained in the study. Of those eligible, 607 individuals (607/1312, 46.3%) randomized to the systematic arm participated in the study.

A total of 1479 participants were listed at the primary care centres in the opportunistic arm. Among these, 89 individuals (6.0%) did not visit the clinic during the study period, leaving 1 390 individuals that could be invited if fulfilling inclusion criteria. Despite training and information, 641 individuals (46.1%) were not assessed for inclusion by the primary care centres (*Figure 2*). Of the 749 individuals who were assessed for eligibility, 140 individuals (18.7%) fulfilled the exclusion criteria, leaving 609 eligible participants. In total, 374 individuals (61.4%) accepted the invitation. Participation rate among invited participants was significantly higher in the opportunistic arm compared with the systematic arm (61.4% vs. 46.3%, *P* < 0.005), whereas population reach was significantly lower (374/1479, 25.3% vs. 607/1437, 42.2%; *P* < 0.005).

**Figure 3:**
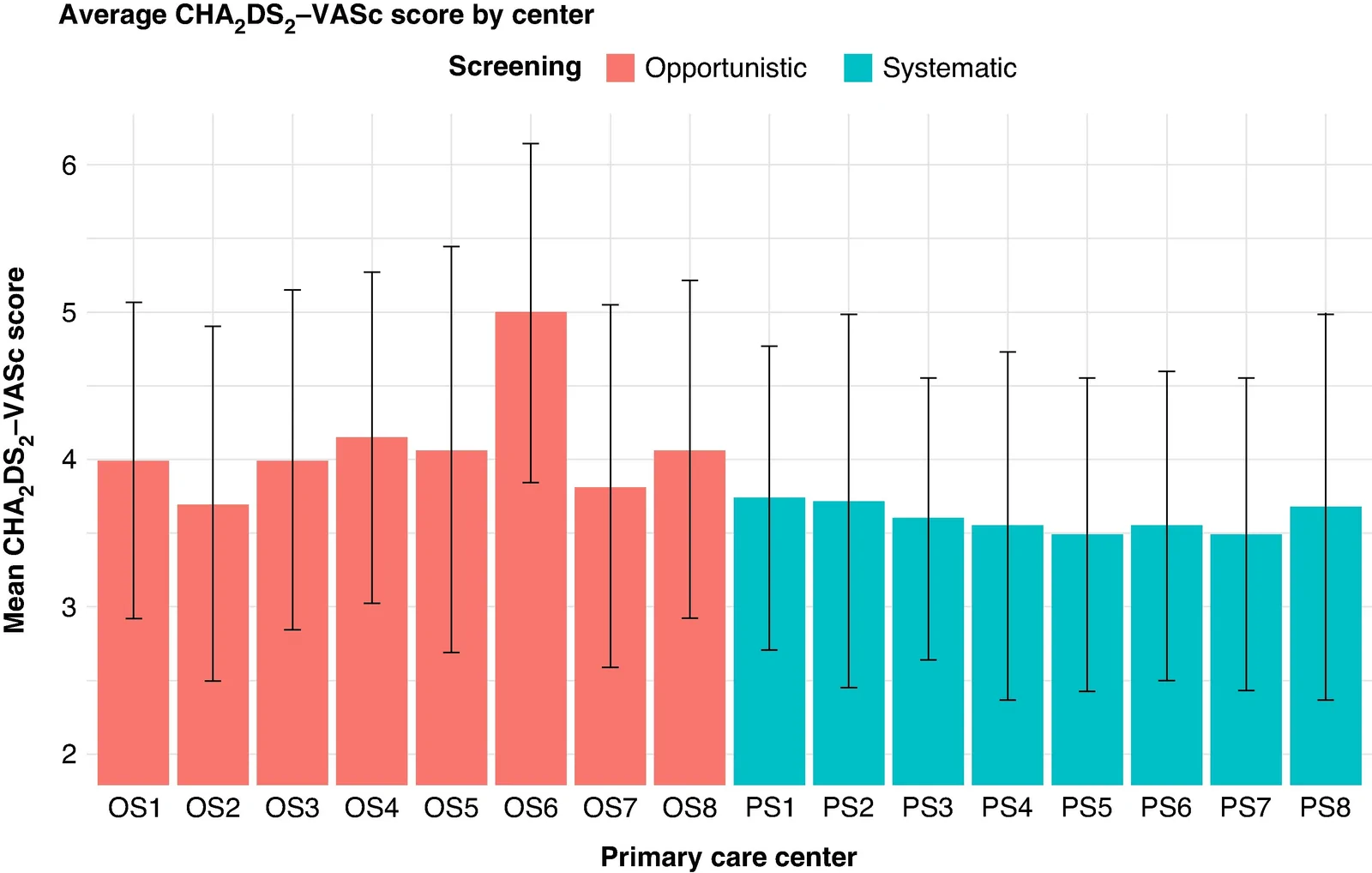
Bar chart of average CHA_2_DS_2_-VASc score compared between the different centres belonging to systematic screening and opportunistic screening. OS, opportunistic screening; PS, population-based screening.

Participants in the opportunistic arm had a higher burden of cardiovascular risk factors, including more cases of diabetes and hypertension, compared with participants in the systematic arm, as shown in *Table 1* (*P* < 0.005). Accordingly, the mean CHA_2_DS_2_-VASc score was higher in the opportunistic arm compared with the systematic arm (3.99 vs. 3.60, *P* < 0.005), with most centres showing higher mean scores among invited participants (*Figure 3*).

**Table 1:** Clinical characteristics of participants in systematic arm and opportunistic arm

	Systematic arm	Opportunistic arm	*P*-value
Accepted invitation	607	374	<0.005
Female (%)	298 (49.1%)	200 (53.5%)	0.21
Clinical risk factors			
Hypertension, *n* (%)	375 (61.8%)	289 (77.3%)	<0.005
Heart failure, *n* (%)	19 (3.1%)	18 (4.8%)	0.24
COPD, *n* (%)	30 (4.9%)	23 (6.1%)	0.50
Diabetes, *n* (%)	99 (16.3%)	90 (24.1%)	<0.005
Valvular disease, *n* (%)	24 (4.0%)	7 (1.8%)	0.10
Hyperthyroidism, *n* (%)	3 (0.5%)	3 (0.8%)	0.68
CKD, *n* (%)	12 (2.0%)	11 (2.9%)	0.45
RA, *n* (%)	12 (2.0%)	10 (2.7%)	0.62
Gout, *n* (%)	23 (3.8%)	19 (5.1%)	0.42
Vascular disease, *n* (%)	81 (13.3%)	67 (17.9%)	0.06
Vascular event/TIA/stroke, *n* (%)	51 (8.4%)	40 (10.7%)	0.28
Mean CHA_2_DS_2_-VASc-Score (SD)	3.60 (1.1)	3.99 (1.2)	<0.005

COPD, chronic obstructive pulmonary disease; CKD, chronic kidney disease; RA, rheumatoid arthritis; TIA, transient ischaemic attack; SD, standard deviation.

Exclusion criteria were more frequently fulfilled in the opportunistic arm **(**18.7% vs. 8.7%, *P* < 0.005) as shown in *Table 2*. AF accounted for most of the fulfilled exclusion criteria in both arms and was significantly increased in the systematic arm compared to the opportunistic arm (84.0% vs. 60.0%, *P* < 0.005), while cognitive impairment was more common in the opportunistic arm (17.9% vs. 0.8%, *P* < 0.005), as shown in *Table 2*. The proportion of patients receiving OAC therapy was similar between groups (13.6% vs. 11.4%, *P* = 0.73).

**Table 2:** Causes of exclusion in the opportunistic arm compared to systematic arm

	Systematic arm	Opportunistic arm	*P*-value
Number of participants assessed for inclusion	1437	749	N/A
Fulfiled exclusion criteria, *n* (%)	125 (8.7%)	140 (18.7%)	<0.005
AF, *n* (%)	105 (84.0%)	84 (60%)	<0.005
OAC, *n* (%)	17 (13.6%)	16 (11.4%)	0.73
Cognitive impairment, *n* (%)	1 (0.8%)	25 (17.9%)	<0.005
Inability to perform ECG at home, *n* (%)	2 (1.6%)	0 (0%)	0.222
Number of AF cases detected	9 (1.5%)	4 (1.1%)	0.77

AF, atrial fibrillation; OAC, oral anticoagulation.

Newly detected AF was numerically low and did not differ between groups (9 vs. 4, *P* = 0.77). The proportion of patients assessed for inclusion varied substantially across opportunistic centres (median 47.0%, IQR 37.0–70.5), while participation among screened individuals was more consistent (median 59.4%, IQR 58.4–68.2) (*Table 3*). Notably, most attrition in the opportunistic arm occurred before invitation: among 1390 clinic attendees, 641 (46.1%) were not assessed. By contrast, among eligible assessed individuals, only 235 of 609 (38.6%) did not participate after screening was offered.

**Table 3:** Screening cascade across opportunistic screening centres

OS centre	Residents listed, *n*	Residents without a visit to the clinic, *n* (%)	Patients assessed for inclusion, *n* (%)	Patients with exclusion criteria, *n* (%)	Participated in the study, *n* (%)
OS 1	274	16 (5.8%)	228 (88.4%)	51 (22.3%)	105 (59.3%)
OS 2	188	8 (4.3%)	80 (43.4%)	12 (15.0%)	40 (58.8%)
OS 3	97	6 (6.2%)	46 (50.5%)	3 (6.5%)	28 (65.1%)
OS 4	113	8 (7.1%)	44 (41.9%)	12 (27.2%)	19 (59.4%)
OS 5	245	14 (5.7%)	74 (32.0%)	22 (29.7%)	39 (75.0%)
OS 6	168	14 (8.3%)	15 (9.7%)	3 (20.0%)	4 (33.3%)
OS 7	132	15 (11.4%)	82 (70.1%)	16 (19.5%)	47 (71.2%)
OS 8	262	8 (3.1%)	180 (70.9%)	21 (11.67%)	92 (57.9%)
Total	**1479**	**89** (**6.0%)**	**749** (**53.9%)**	**140** (**18.7%)**	**374** (**61.4%)**

The table shows the number of participants listed at each centre, those without a clinic visit during the study period, individuals assessed for eligibility, those exclusion criteria, and final participation.

On average, the participants performed a mean of 42.4 ECG recordings, exceeding the expected number of recordings (three per day over 14 days), indicating excellent adherence. There was no statistically significant difference in the number of ECGs performed between the groups randomized to systematic vs. opportunistic screening (*P* = 0.12).

## Discussion

The STROKESTOP III study compared opportunistic screening for AF with systematic-based screening in individuals aged 75–76 years. The main finding is that opportunistic screening achieved a significantly higher participation rate compared with systematic screening (61.4% vs. 46.3%, *P* < 0.005). However, this higher uptake did not translate into higher population reach, because 46.1% of clinic attendees in the opportunistic arm were not assessed for eligibility, resulting in lower overall reach compared with systematic screening (25.3% vs. 42.2%). Thus, opportunistic screening showed high individual level acceptance when offered but was substantially limited by implementation barriers.

Participation in the systematic arm was similar to that reported in previous population-based AF screening trials, including STROKESTOP I and STROKESTOP II, where approximately half of the invited individuals participated.^10,12,17^ Hence, participation in population-based AF screening programmes has remained relatively stable over time despite increased awareness of AF and stroke prevention. In contrast, participation rates were higher when screening was offered opportunistically during routine healthcare encounters. Screening offered directly by healthcare staff may be perceived as more relevant and requires less initiative from the individual compared with responding to a mailed invitation. Public awareness of AF remains limited, with recent surveys demonstrating important knowledge gaps regarding AF and its consequences.^20^ Healthcare encounters may therefore provide not only an opportunity for screening but also an opportunity for patient education. Broader public awareness initiatives combined with opportunistic screening may increase willingness to participate in future screening programmes.

Participants recruited through opportunistic screening had a higher burden of cardiovascular risk factors. Hypertension and diabetes were more common, and the mean CHA_2_DS_2_-VASc score was higher in the opportunistic arm (3.99 vs. 3.60, *P* < 0.005). This supports the notion that opportunistic screening preferentially reaches individuals with more frequent contact with the healthcare system and therefore potentially higher baseline risk of stroke.

Fulfilment of exclusion criteria was more frequent in the opportunistic arm, primarily due to previously diagnosed AF and cognitive impairment. This likely reflects that individuals presenting to primary care represent a more comorbid population with distinct healthcare-seeking behaviour and are therefore more likely to meet exclusion criteria or already have diagnosed AF prior to screening. The lower proportion of cognitive impairment in the population-based arm may reflect underreporting, as exclusions were self-reported, whereas in the opportunistic arm cognitive impairment could be identified directly by the clinic.

We also observed substantial variation between centres in the opportunistic arm in the proportion of individuals assessed for eligibility for participation. This suggests that the effectiveness of opportunistic screening depends strongly on local implementation and available clinical resources. Opportunistic screening requires healthcare personnel to identify eligible patients during routine visits, which may compete with other clinical priorities. Notably, nearly 46% of individuals in the opportunistic arm who attended primary care during the study period were not assessed for eligibility, suggesting considerable unrealized screening potential. This proportion might potentially be reduced with greater personnel support or dedicated screening resources. The high non-assessment rate is central to interpreting the opportunistic strategy. In practice, the study required staff to identify an eligible patient during a routine visit, check exclusion criteria, explain the study, and invite the patient within the ordinary clinical workflow. The large proportion of clinic attendees who were not assessed likely reflects limited staff time and competing clinical priorities rather than patient unwillingness. Importantly, this non-assessment category was not anticipated as a major attrition step in the study design paper, and it should therefore be regarded as a key implementation outcome of the present trial. Non-assessment may also have introduced selection bias because staff may have been more likely to approach patients with more frequent healthcare contact, more evident cardiovascular comorbidity, or those perceived as suitable for an additional screening discussion. A comparable approach was used in the SAFER trial, which combined electronic health record–based identification of eligible individuals with centralized postal distribution of handheld ECG devices; a similar model could reduce opportunistic screening's dependence on in-person staff availability.^21^

These findings may have important implications for AF screening implementation. Although handheld ECG devices are user-friendly and feasible for screening,^22^ our results suggest that personnel time and workflow integration may represent greater barriers than the ECG screening itself. As AF screening remains incompletely implemented in many healthcare systems,^23^ dedicated resources or automated identification of eligible individuals may be needed to improve uptake.

While the SAFE study suggested that opportunistic screening may be comparable to systematic screening for AF detection while potentially being cost-effective, our findings extend these observations by highlighting a critical implementation challenge.^16^ Although participation among invited individuals was higher with opportunistic screening, overall reach depended heavily on successful execution at the primary care level, suggesting that the effectiveness of opportunistic screening may be closely linked to available infrastructure and clinical resources.

Despite the higher cardiovascular risk profile among participants in the opportunistic arm, AF detection did not differ significantly between the two screening strategies. The total number of newly detected AF cases was low, with only 13 cases identified during the study. One possible explanation is that AF detection in routine clinical care has increased over recent years. National prescription data show a marked rise in OAC use among older individuals, suggesting that more patients with AF are already diagnosed and treated before reaching the age at which screening is offered (see Supplementary material online, *Figure S1*).

In summary, our findings highlight complementary strengths and limitations of the two screening approaches. Opportunistic screening achieved higher participation among those invited and reached a population with a higher cardiovascular risk profile. However, it depended heavily on successful implementation of screening protocols at individual primary care centres and reached fewer individuals overall. Systematic screening, in contrast, ensures broader population coverage but is limited by lower participation and a potential healthy participant bias.

Future AF screening programmes may therefore benefit from combining these approaches. Systematic invitations could provide broad coverage of the target population, while opportunistic screening during routine healthcare visits may help identify high-risk individuals who might otherwise not respond to population-based invitations. Emerging artificial intelligence-based prediction models may further improve the efficiency of AF screening programmes by identifying individuals with the highest risk of undetected AF. Recent studies have demonstrated the feasibility of combining risk prediction algorithms with ECG-based screening strategies to improve screening for AF detection.^13,24^

### Limitations

The study had several limitations. Only 16 centres were included in the cluster randomization, which could influence both patient characteristics and participation rate. This was reflected in the wide variation in invitation rate in opportunistic centres. The study was not powered for AF detection, and the low number of participants diagnosed with AF limits conclusions regarding which screening strategy is most effective for identifying previously undiagnosed AF.

## Conclusion

In conclusion, STROKESTOP III shows that opportunistic screening achieved high participation when individuals were actively approached in routine care and tend to engage a higher-risk population, although its overall population reach remains limited mainly due to many individuals not being assessed for eligibility. Systematic screening offers broader coverage but is constrained by lower participation and healthy selection bias. Future strategies should aim to combine high reach with high participation.

## Supplementary Material

euag214_Supplementary_Data

## Data Availability

The data that support the findings of this study are not publicly available due to privacy and ethical concerns, as they contain sensitive patient information. Access to the data is restricted in accordance with patient confidentiality agreements and applicable regulations. Researchers interested in the data for replication or further study may contact the corresponding author, but any access will be subject to appropriate data use agreements and ethical approvals.
