# Supplementary material for: Population reach and participation rate in opportunistic vs. systematic atrial fibrillation screening: STROKESTOP III
Source: Europace. 2026 Aug 30;28(8):euag214. doi: 10.1093/europace/euag214 (PMC13526252; doi:10.1093/europace/euag214)
Supplement: Supplementary file 1 [file euag214_supplementary_data.docx]

**Supplementary figure 1.** Supplementary Figure 1. Annual number of individuals aged 75–76 years prescribed oral anticoagulants (OAC) in Region Värmland, Sweden, from 2011 to 2024, according to data from the Swedish National Board of Health and Welfare (Socialstyrelsen).
